# Histomorphometric Analysis of Osseointegrated Intraosseous Dental Implants Using Undecalcified Specimens: A Scoping Review

**DOI:** 10.3390/biomimetics9110672

**Published:** 2024-11-03

**Authors:** Stefan Peev, Ralitsa Yotsova, Ivaylo Parushev

**Affiliations:** 1 Department of Periodontology and Dental Implantology, Faculty of Dental Medicine, Medical University of Varna, 9002 Varna, Bulgaria; stefan.peev@mu-varna.bg; 2 Department of Oral Surgery, Faculty of Dental Medicine, Medical University of Varna, 9002 Varna, Bulgaria; 3 Department of Clinical Medical Sciences, Faculty of Dental Medicine, Medical University of Varna, 9002 Varna, Bulgaria; ivaylo.parushev@mu-varna.bg

**Keywords:** histology, histomorphometry, undecalcified, non-decalcified, dental implant, osseointegration

## Abstract

Bone histology and histomorphometry are reliable diagnostic tools for the assessment of the bone–implant interface, material safety and biocompatibility, and tissue response. They allow for the qualitative and quantitative analysis of undecalcified bone specimens. This scoping review aims to identify the most common staining techniques, study models for in vivo experiments, and histomorphometric parameters used for quantitative bone evaluation of osseointegrated dental implants in the last decade. The Web of Science, PubMed, and Scopus databases were searched on 1 July 2024 for relevant articles in English, published in the last ten years, and the data were exported to an MS Excel spreadsheet. A total of 115 studies met the eligibility criteria and were included in the present review. The results indicate that the most common study models are dogs, rabbits, and pigs. Some of the most frequently used methods for the assessment of the bone–implant interface are the Toluidine blue, Stevenel’s blue with Van Gieson, and Levai–Laczko stainings. The results from this study demonstrate that the most commonly used histomorphometric parameters in implant dentistry are the bone-to-implant contact (BIC), bone area fraction occupancy (BAFO), bone area (BA), and bone density (BD). This review presents the recent trends in histomorphometric analysis of dental implants and identifies some research gaps that necessitate further research.

## 1. Introduction

Replacing missing dental tissue with prosthetic constructions supported by root-form implants is a biomimetic approach to rehabilitating the dentition impaired by pathological processes. Dental implant placement is a routine procedure that has been widely recognized as a treatment modality of choice for restoring missing teeth [1]. Intraosseous dental implants made of commercially pure titanium are still regarded as the gold standard [2] and are the most commonly used dental implants due to their high success rate of up to 99% [3,4]. The alternatives to titanium implants include implants made of titanium alloys, zirconia, a combination of titanium and zirconia, and implants with various coatings, which have demonstrated excellent tissue behavior and survival rates [5,6,7,8].

The foundation of contemporary dental implantology was laid by Brånemark et al. [9,10], who found evidence of direct bone apposition on implant surfaces—a phenomenon that was later referred to as “osseointegration”.

Osseointegration is a biomimetic rather than a physiological process. It was first defined as “direct contact between a loaded implant surface and bone at a light microscopic level” [11]. It was later defined as the “clinically asymptomatic rigid fixation of alloplastic materials”, which is “achieved and maintained in bone during functional loading” [12].

As described by John Davis, the third phase of contact osteogenesis is a biomimetic process resembling bone remodeling [13]. In this sense, the analysis of the parameters that are the subject of the present study represents a biomimetic process replicating the bone remodeling triggered by trauma.

Osseointegration is a dynamic process initiated during implant placement, with primary implant stability leading to secondary stability [14]. It is still unknown to what extent an implant should be in direct contact with vital bone to be regarded as “osseointegrated”. There are no exact criteria for the evaluation of this process. Moreover, bone is a vital and dynamic tissue that undergoes constant remodeling [15,16]. Therefore, it requires not only static but also dynamic assessment.

It has become evident that implant material, design, and surface characteristics have key impacts on osseointegration success. In the last decades, various biomaterials, implant designs, and coatings have been developed and evaluated in order to improve long-term treatment results [17,18,19]. Furthermore, numerous analytical techniques and clinical and paraclinical tests have been utilized for the assessment of the osseointegration process and implant stability, such as torque tests, resonance frequency analysis, fractal analysis, computed tomography (CT), cone-beam CT, micro-CT, scanning electron microscopy, transmission electron microscopy, Raman spectroscopy, histological evaluation, histomorphometric analysis, immunohistochemistry, and so on [20,21,22,23,24]. It has been evident that implant material, design, and surface characteristics have key impacts on osseointegration success. In the last decades, various biomaterials, implant designs, and coatings have been developed and evaluated in order to improve long-term treatment results [17,18,19].

Light optical microscopy is a commonly utilized diagnostic tool for the observation of biopsy specimens and the evaluation of material behavior and tissue responses. It allows for both qualitative (histological) and quantitative (histomorphometric) assessment. Qualitative histology provides a detailed description of tissues and their morphology, maturity, and cellular and non-cellular components [25,26,27].

Bone histomorphometry is the gold standard for the evaluation of bone density, implant stability and osseointegration, material behavior, and tissue response [13,28]. Until the middle of the 20th century, the histological evaluation of bone necessitated its prior decalcification. The introduction of plastic embedding allowed for a more precise assessment of undecalcified bone specimens [27,29].

Histomorphometric assessment can be divided into two major groups: static and dynamic histomorphometry. Examples of static histomorphometric parameters are bone-to-implant contact (BIC), bone density (BD), and the amount and characteristics of the cellular content, whereas examples of dynamic parameters are mineral apposition rate and fluorescence analysis [30,31,32]. Dynamic parameters are evaluated after fluorochrome labeling, which allows for the visualization of bone remodeling [32,33,34].

The most commonly evaluated histomorphometric parameter is BIC [35]. It is described as “the percentage of implant surface in direct contact with bone tissue” [13,36]. However, numerous studies do not specify what type of bone (new, old, or total) covers the implant when calculating BIC. In addition, it is still not clear if BIC is informative enough to serve as a marker of implant stability [37], although it is used for the evaluation of the speed of new bone deposition. It is a well-known fact that implant stability depends on several factors, including the percentage of BIC, the type of connection between the new bone deposited on the implant surface and the surrounding bone, and bone density [13,38].

The region of interest (ROI) is not a strictly defined term and depends on the evaluated parameters and the research objectives. For instance, when BIC is assessed, the ROI can trace the whole implant perimeter and the areas that demonstrate bone deposition on the implant surface (%BIC = length of BIC/total perimeter length of the implant) [31,39,40,41]. In contrast, some studies use only one implant thread at a time as an ROI [42,43,44].

Numerous staining methods have been developed and introduced, and most of them allow for excellent histological evaluation [28]. However, these methods tend to stain different structures in similar colors and shades, which makes computer-assisted assessment extremely challenging. Although hand tracing is possible in such cases, the process is labor-intensive and time-consuming [36]. Toluidine blue has been reported as the most common staining technique for the histological evaluation of dental implant osseointegration [35].

Microscopic observation of the interaction between bone, bone substitutes, and dental implants through animal experiments has become a common research strategy for the evaluation of material behavior and cellular and tissue responses.

Animals are commonly used as in vivo models in biomedical and biomaterials sciences due to the impossibility of such experiments on humans. The selection of appropriate animal species that resemble human biology, physiology, and pathophysiology is a crucial criterion for obtaining results that are transferable to clinical applications [25,45].

Various animal models have been used in the existing scientific literature for in vivo testing of implant osseointegration, material behavior, safety and efficacy, and the tissue response to biomaterials, implants, and coatings [14,46]. These studies, however, present a high degree of heterogeneity due to interspecies differences, different work protocols, implant characteristics, loading conditions, and so on. Therefore, a direct comparison between them is not feasible [14].

It has been suggested that irrespective of the animal model (dog, rat, rabbit, pig, goat, sheep, or monkey) or the selected surgical site, studies can provide valuable results when properly designed [30].

The present review discusses the histomorphometric assessment of osseointegrated intraosseous dental implants using undecalcified bone specimens. It aims to identify the most frequently used staining methods, study models, and histomorphometric parameters (study variables) for the histomorphometric analysis of intraosseous dental implants in recent years. The study analyzes their advantages and limitations, identifies some research gaps, and gives recommendations for further research. To the best of our knowledge, this is the first scoping review that identifies the recent trends in the histomorphometric analysis of dental implants regarding these variables.

Implant dentistry is a rapidly developing field and, recently, researchers have been focused on introducing new biomimetic materials, scaffolds, surface modifications, and implant coatings [47,48,49,50]. Their accurate evaluation requires appropriate and reliable histological and histomorphometric protocols. The results of this study indicate a lack of unification of the specific terminology and clear guidelines for applying the existing protocols for histomorphometric observations, which is a major drawback, necessitating further research and evaluation.

## 2. Materials and Methods

This scoping review adhered to the PCC framework with the following components.

**P (Patient/Population/Participants)**—Study models (human and animal) used for the evaluation of dental implant osseointegration

**C (Concept)**—Types of staining techniques and histomorphometric parameters

**C (Context)**—Research articles published in the last 10 years (2014–2024)

**Review Question:** What types of staining techniques and histomorphometric parameters (C) are used in the different study models (human and animal) for the evaluation of dental implant osseointegration (P) in the last 10 years (C)?

**Eligibility Criteria**

The inclusion criteria were as follows: original research articles; articles that evaluated dental implant osseointegration using undecalcified plastic-embedded specimens; and articles that reported the study models and histological stainings used.

The exclusion criteria were as follows: 1. review articles, books, book chapters, and abstracts; 2. articles that did not discuss undecalcified plastic-embedded specimens; 3. articles that did not include histological staining; 4. articles that did not discuss histomorphometric parameters; 5. articles that did not discuss osseointegrated intraosseous dental implants; 6. articles from before 2014.

**Information Sources**

This scoping review was conducted in accordance with the Preferred Reporting Items for Systematic Reviews and Meta-Analysis for Scoping Reviews (PRISMA-ScR) [51]. It was registered in the Open Science Framework (OSF) and can be found at https://archive.org/details/osf-registrations-9b8yx-v1 (accessed on 12 July 2024).

A comprehensive electronic search for research articles in the Web of Science, PubMed, and Scopus databases was conducted on 1 July 2024.

**Search Strategy**

The search strategy comprised an advanced search in the selected databases using combinations of keywords and MeSH Terms. It encompassed articles published in the last 10 years to capture the most up-to-date evidence on the topic and present the recent trends in the field.

1. The keywords that were used in the Web of Science database were: ALL = (((histology) OR (histomorphometry)) AND ((undecalcified) OR (non-decalcified)) AND (dental implant) AND (osseointegration)).
2. The keywords that were used in the PubMed database were: ((histology) OR (histomorphometry)) AND ((undecalcified) OR (non-decalcified)) AND (dental implant) AND (osseointegration).
  The expanded query string was: (“anatomy and histology” [MeSH Subheading] OR (“anatomy” [All Fields] AND “histology” [All Fields]) OR “anatomy and histology” [All Fields] OR “histology” [All Fields] OR “histology” [MeSH Terms] OR “histologies” [All Fields] OR “histomorphometry” [All Fields]) AND (“undecalcified” [All Fields] OR “non-decalcified” [All Fields]) AND (“dental implants” [MeSH Terms] OR (“dental” [All Fields] AND “implants” [All Fields]) OR “dental implants” [All Fields] OR (“dental” [All Fields] AND “implant” [All Fields]) OR “dental implant” [All Fields]) AND (“osseointegrate” [All Fields] OR “osseointegrated” [All Fields] OR “osseointegrates” [All Fields] OR “osseointegrating” [All Fields] OR “osseointegration” [MeSH Terms] OR “osseointegration” [All Fields] OR “osseointegrative” [All Fields]).
3. The keywords that were used in the Scopus database were: ((histology) OR (histomorphometry)) AND ((undecalcified) OR (non-decalcified)) AND (dental AND implant) AND (osseointegration) AND PUBYEAR > 2013 AND PUBYEAR < 2025 AND (LIMIT-TO (DOCTYPE, “ar”)) AND (LIMIT-TO (LANGUAGE, “English”)).

**Study Selection, Data Collection, and Data Items**

The titles, abstracts, author names, and years of publication of the studies were exported to an MS Excel spreadsheet. Then, duplicate records were removed. Titles and abstracts were screened and evaluated for eligibility by two independent reviewers (R.Y. and I.P.) and then the full-text studies were subjected to the inclusion and exclusion criteria. The data retrieved from each article included authors and year of publication, study models, sample size, implant material, healing time, staining technique, augmentation (if performed), and histomorphometric parameters. In addition, the definitions of ROI were extracted, if present. Discrepancies among the reviewers were resolved through discussion, consensus, and arbitration by a third reviewer (S.P.) when a consensus between the first two was difficult to reach.

Synthesis Methods

Each study was evaluated by two independent reviewers (R.Y. and I.P.) and the data were synthesized in tables in MS Excel Spreadsheets. Decisions about articles with missing data were made through discussion until a consensus was reached.

## 3. Results

The initial search identified 294 potentially relevant studies from the three electronic databases over the last ten years. After the exclusion of 29 duplicate records, 265 articles remained. Then, the articles that did not meet the eligibility criteria were excluded. Finally, a total of 115 studies were included in the present review.

**Figure 1:**
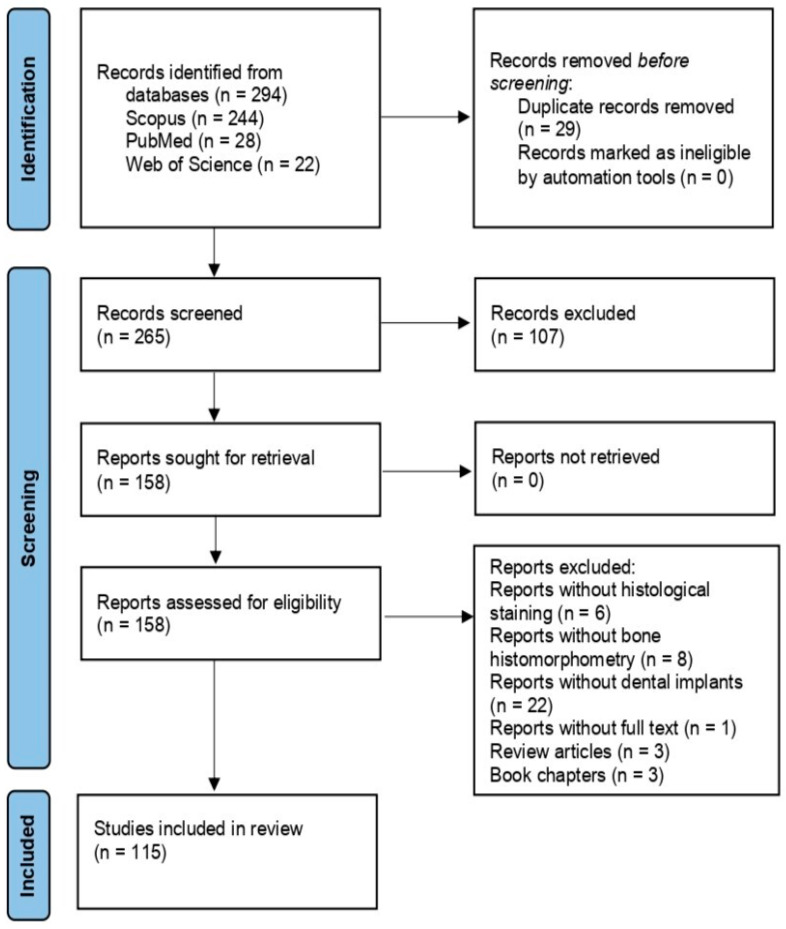
PRISMA flow diagram of the research [52].

Figure 1 illustrates a PRISMA flow chart of the selection process [52].

Table 1 presents the characteristics of the studies included in this scoping review.

**Figure 2:**
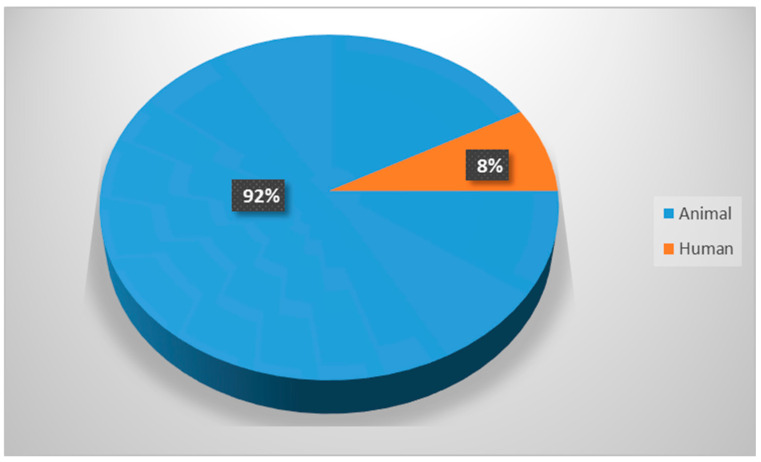
Distribution of animal and human trials.

**Figure 3:**
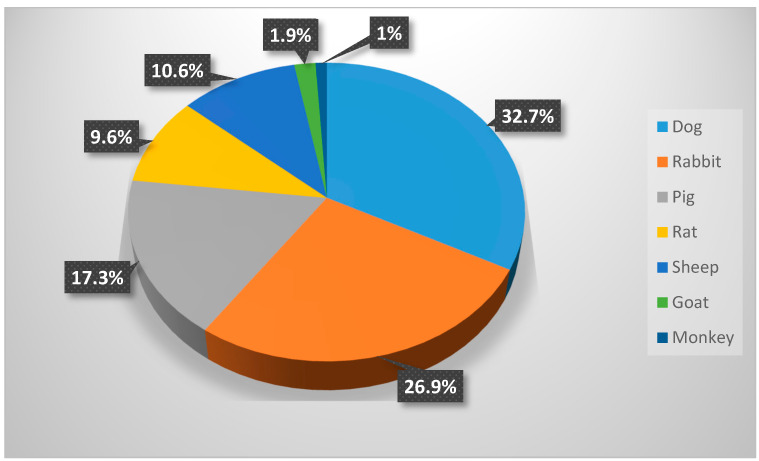
Distribution of animal experiments.

The study results demonstrated that most of the in vivo experiments utilized animal rather than human models (Figure 2), with dog, rabbit, and pig models being the most commonly used (in more than 70% of the cases), followed by rat, sheep, goat, and monkey models (Figure 3).

**Figure 4:**
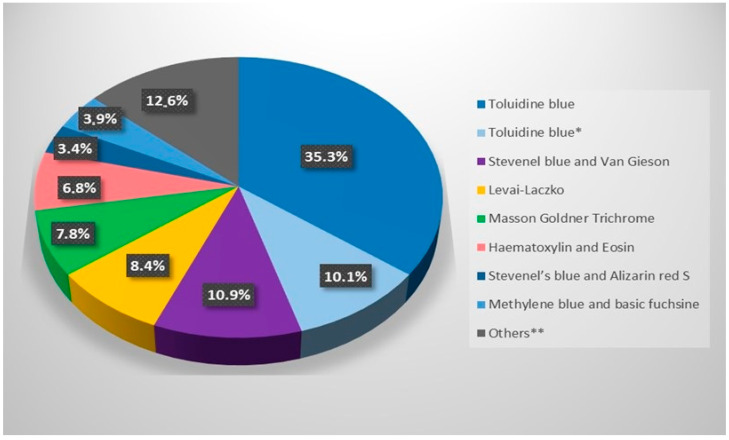
Histological staining techniques for bone histomorphometric analysis of osseointegrated intraosseous dental implants. * Toluidine blue in combination with other dyes such as pararosaniline, pyronin, fast green, basic fuchsin, and Giemsa solution ** van Gieson, Alizarin red, Methylene blue-Alizarin red, MOVAT Pentachrome (after Verhöff), Fibrin stain of Ladewig, Picro-syrius-Hematoxylin, Basic fuchsin and Light green, Hematoxylin-Eosin-Safran, Sanderson’s RBS stain and acid fuchsin.

As for the staining methods, the most commonly used were toluidine blue (alone or with different counterstains), Stevenel blue with Van Gieson, and Levai–Laczko (Figure 4).

This scoping review indicates that the most frequently evaluated histomorphometric parameters were BIC (in more than 90% of studies), BAFO, BA, and BD. The definitions of BAFO, BA, and BD, however, suggest that they are usually equivalent terms that describe the ratio between the area occupied by bone and the total area. The only difference seems to be the type of bone area considered. Some authors considered the whole bone area [57,59,60,64,69,87,90,92,103,104], whereas others considered only mineralized bone [76,80,81,88,97,98].

The region of interest is a specific zone that is the target of assessments and analysis. Some authors clearly defined the ROI, while others did not specify what ROI was used in their research.

Table 2 shows the definitions of the ROIs from the studies included in this review, when present, as well as the observed histomorphometric parameters.

## 4. Discussion

Animal testing has been regarded as a reliable method for biomedical research and a prerequisite to human clinical trials. Animals are used as in vivo models in order to obtain results for translation to clinical settings. This requires a precise selection of animal models based on their resemblance to the human organism. In the fields of biomedicine and biomaterials science, animal models are used to reproduce the biological and physiological pathways of humans. However, animal species differ significantly from humans regarding their anatomical features and physiological reactions. Therefore, a careful selection of the most appropriate animal model for each study is required [45].

A wide range of species has been used for these purposes through the years, from small laboratory rodents to larger animals such as dogs, pigs, and non-human primates [32,44,45]. By mimicking the biological environment in humans, pre-clinical trials allow for the investigation of the interaction between implants and peri-implant tissues. All animal models have some advantages and disadvantages. However, none of them can sufficiently represent all levels (macro, micro, and nano) of implant osseointegration [45]. It has been reported that the most commonly used animal models for implant biomaterial research are dogs, sheep, pigs, rabbits, goats, and rodents [31,168]. According to the findings in this scoping review, dogs are the most frequently used animals, followed by rabbits, pigs, rats, sheep, goats, and monkeys.

Dog models are often preferred due to their larger size and the resemblance of their bone morphology and mineral density to those of humans. A major drawback is the variable inter-individual and intra-individual trabecular bone turnover [33].

Rabbit models are cost-effective, non-aggressive, and easy to house and handle. They reach their skeletal maturity at 6 months, which allows for fast experimental results. However, their rapid bone remodeling and turnover differ significantly from those of human bones [33].

Pigs as animal models have numerous advantages, such as their size, well-developed Haversian systems, and similar bone mineral density and bone mineral concentration to those of humans. In addition, they demonstrate similar bone remodeling processes and bone regeneration rates. However, they are more aggressive and difficult to handle [33].

Rat models are cost-effective and easy to handle. Their major drawbacks are their small size, which provides a limited surgical area; rapid metabolic rate; and higher osteogenic potential than that of humans. Furthermore, their bone morphology and mineral density differ from those of humans [33].

Sheep have similar bone turnover and remodeling rates to those of humans. However, their bone microstructure differs significantly from that of humans. Moreover, sheep undergo seasonal periods of bone resorption [33].

Non-human primates have been utilized due to their morphological and genetic resemblance to humans. Recently, they have almost stopped being used for such purposes due to ethical reasons and housing difficulties [46].

In recent years, advances in biomedical research have led to the progressive replacement of animal testing by using in vitro experiments and biomaterial strategies, thus avoiding animal sacrifice.

In 2011, Lang et al. [169] demonstrated that the process of osseointegration in humans is slower when compared with what had been previously reported in animal models, such as dogs [170] and minipigs [171]. Therefore, the authors concluded that a direct comparison between the results from human and animal studies, using BIC as a marker of osseointegration, is impossible.

Most of the studies included in this review evaluated dental implants made of titanium rather than zirconia, which could indicate that titanium remains the gold standard for the fabrication of dental implants.

Undecalcified bone histology and histomorphometry provide essential information on bone quality and quantity [29]. Plastic embedding allows for the observation and assessment of peri-implant tissues in undecalcified bone specimens [29,168,172]. This method provides the structural preservation and stabilization necessary for static and dynamic histomorphometric evaluation [29,173,174,175,176,177].

Almost all histological staining methods can be used for bone histological and histomorphometric assessment. The results of this review demonstrate that the most commonly used staining techniques for bone histomorphometric analysis of osseointegrated intraosseous dental implants are toluidine blue, Stevenel’s blue with Van Gieson, and Levai–Laczko.

Toluidine blue staining provides clear identification of mineralized bone, osteoid tissue, and soft tissues [29,178,179,180]. Furthermore, this method allows for the observation of osteons and cement lines [29,170,180,181,182,183,184], which mark the line between old and newly formed bone, and has been successfully utilized for the evaluation of the bone–implant interface [29,60,73,185,186,187,188,189]. 

The aim of staining bone specimens is to visualize the healing process, the areas of new bone formation, and the inflammatory response within the defect. The hematoxylin-and-eosin staining technique is usually utilized for the establishment of material safety, while Stevenel’s blue and Goldner’s trichrome stainings can be used for the evaluation of their efficacy. Hematoxylin-and-eosin staining is commonly used for the assessment of inflammatory reactions and biocompatibility [33]. However, it does not allow for a clear distinction between pre-existing and newly formed bone. Therefore, the utilization of additional stains is necessary for the visualization of new bone apposition.

A wide range of staining methods, such as von Kossa, Alizarin Red, Goldner’s trichrome, van Gieson, Stevenel’s blue, Safranin O/Fast green, and so on, can be used for plastic-embedded specimens [32]. Stevenel’s blue with van Gieson’s picrofuchsin as a counterstain provides excellent identification of pre-existing bone and areas of new bone formation. However, the distinction between osteoid and soft tissues can be difficult [33].

Van Gieson staining can be successfully used for the assessment of the bone–implant interface. Stevenel’s blue staining provides a visualization of fibrous tissue [33]. Levai–Laczko staining provides a visualization of the areas of old bone, newly formed bone, and bone-grafting materials.

Quantitative bone histomorphometry requires a distinction between osteoid and mineralized bone. Several staining techniques can be used for this purpose, such as methylene blue basic fuchsin, von Kossa with hematoxylin and eosin counterstaining, Goldner’s trichrome, and Sanderson’s rapid bone with either acid fuchsin or van Gieson counterstaining [33].

Computer-assisted histomorphometric evaluation requires the use of staining protocols that provide different stainings of bone microstructures. Therefore, the “perfect” staining method should allow for an excellent distinction between these units. In addition, it should have the following advantages: reproducibility, ease of use, and cost-effectiveness.

Numerous histomorphometric parameters have been used for the evaluation of implant osseointegration, the bone–implant interface, material behavior, and so on. As previously stated, this review indicates that the most commonly used histomorphometric parameter is BIC, which is calculated as the region of such contact along the implant perimeter subtracted from the total implant perimeter. This parameter has been used as a reliable marker of osseointegration and implant stability [190]. Most authors have suggested similar definitions for BA, BD, and BAFO, and used them to calculate the ratio between the bone area and total area in ROIs.

There are some discrepancies regarding the definitions of the above-mentioned histomorphometric parameters among studies. This requires either unification of the terminology or a clear explanation of the exact protocol and methodology in each study.

The region of interest is the specific target area of evaluation and analysis. As can be seen in Table 2, even when examining the same histomorphometric parameters, researchers have used different ROIs. This is because ROIs are defined individually by authors and depend on the study objectives. Moreover, some authors do not define the ROI they used, which further hinders the interpretation.

A comparison of the studies that have evaluated bone–implant interfaces is difficult due to differences in their methodologies, including related to in vivo models (human and animal), staining techniques, implant types and materials, histomorphometric parameters, and so on. Unification of the terminology used for bone histomorphometric assessment of dental implants could contribute to an easier interpretation and better understanding of the results.

### 4.1. Strengths

To our knowledge, this is the first scoping review which estimates the frequency of use of the three variables: study models, staining techniques, and histomorphometric parameters. It demonstrates the trends in dental implant research for the last 10 years. In addition, some advantages and limitations of the study models, staining methods, and observed parameters are presented descriptively. Furthermore, the study summarizes the various definitions of ROI and the lack of such information in some studies, identifying a significant research gap.

Understanding the histomorphometric evaluation of dental implants is essential for clinicians in the fields, as it answers questions such as: How soon after implantation can the implants carry functional loading and with what magnitude?; What is the impact of surgical trauma on the peri-implant bone and what are the parameters of the postoperative healing process?; What is the influence of the implant macro design and biomaterial, and the modification of its surface topography and chemistry on the peri-implant healing processes, their speed, qualities, and predictability?

### 4.2. Limitations

A limitation of this scoping review is that a risk of bias assessment of the included studies and a meta-analysis of the results were not performed. This is mainly due to the specificity of the review question, as it focuses on the frequency of use and trends in the field and compares the methodologies used in the studies, not their outcomes. Furthermore, the results of this review show the recent trends but do not explain the rationale for them.

The timeframe of 10 years was selected in this review in order to filter the state-of-the-art protocols for histomorphometric evaluation of dental implant osseointegration and identify the contemporary approaches in the field. Changes in the ethical requirements through the years and the efforts towards reducing the use of animal models have influenced the choice of study models. In addition, the introduction of different biomimetic approaches, including new implant coatings and other implant surface modifications, has led to a growing number of studies on the topic, summarizing the previous knowledge and presenting the most recent protocols. As Babuska et al. indicated, developing biomimetic implant surfaces that resemble natural tissues is an important research topic [35].

The paraffin histology widely used in the past poses the risk of tissue impairment during decalcification. Moreover, embedded hard objects like implants are usually detached from the tissues during paraffin sectioning.

The application of resins as embedding media greatly facilitates the histological and histomorphometric studies of osseointegrated dental implants [35].

### 4.3. Future Directions

Further research, including randomized clinical trials, systematic reviews, and meta-analyses, is necessary to define the most appropriate protocols for the histomorphometric evaluation of osseointegrated intraosseous dental implants. Unification of the histomorphometric terminology and clear guidelines for using the different histomorphometric parameters are required.

## 5. Conclusions

This scoping review identifies the most frequently used staining method (toluidine blue), study model for in vivo experiments (dog), and histomorphometric parameters (BIC) for the assessment of the osseointegration of dental implants in the past 10 years. The study demonstrates the recent trends in the field rather than the scientific rationale for their use.

Unification of the terminology with clear guidelines for using the different histomorphometric parameters and optimization of the suggested protocols are necessary for easier interpretation and better understanding.

## Figures and Tables

**Table 1:** Articles included in the present study.

№	Reference	Authors	Year	Study Model	Sample Size	Implant Material	Healing Time	Staining Method	Augmentation	Histomorphometric Parameters
1	[53]	Adam et al.	2014	Rabbit	36	Titanium	2, 4, 6 weeks	Toluidine blue	No	BIC
2	[54]	Alayan et al.	2017	Sheep	16	Titanium	2, 4 weeks	Toluidine blue	Yes	BIC, WB, LB, RG, STM
3	[55]	Alenezi et al.	2019	Rat	96	Titanium	2, 6 weeks	Toluidine blue-pyronin	No	BIC, BA, new-BA
4	[56]	Almohandes et al.	2019	Dog	24	Titanium	24 weeks	Toluidine blue and fibrin stain of Ladewig	Yes No	BIC
5	[57]	Al-Omari et al.	2020	Rabbit	80	Titanium	4, 6, 8, 10 weeks	Toluidine blue	No	BIC, BAF, Ct.Th
6	[58]	Alshehri et al.	2019	Goat	36	Titanium	7 weeks	Methylene blue and basic fuchsine	Yes No	PIBA
7	[59]	Baires-Campos et al.	2015	Dog	40	Titanium	2, 4 weeks	Toluidine blue	No	BIC, BAFO
8	[60]	Beutel et al.	2016	Dog	72	Not stated	3, 5 weeks	Toluidine blue	No	BIC, BAFO
9	[61]	Boldeanu et al.	2022	Dog	5	Titanium	11 months	Masson Goldner Anilin blue; MOVAT Pentachrome (after Verhöff)	No	imp-BC, IS-BC, IS-BD, BC-BD
10	[62]	Boldeanu et al.	2023	Dog	5	Titanium	11 months	Masson Goldner Anilin blue; MOVAT Pentachrome (after Verhöff)	No	imp-BC, IS-BC, IS-BD, BC-BD
11	[63]	Botzenhart et al.	2015	Minipig	15	Titanium	8 weeks	Masson-Goldner-trichrome	No	BIC
12	[64]	Bowers et al.	2016	Sheep	24	Titanium	6 weeks	Stevenel’s Blue and Van Gieson´s Picro Fuschin	No	BIC, BAFO
13	[65]	Calvo-Guirado et al.	2015	Dog	48	Titanium	30, 90 days	Toluidine blue	No	BIC, CBL
14	[66]	Calvo-Guirado J.	2015	Dog	48	Titanium	12 weeks	Toluidine blue	No	BIC, CBL
15	[67]	Chien et al.	2014	Dog	12	Titanium	2 months	Stevenel blue and Van Gieson picro fuchsin	No	BIC
16	[68]	Chiu et al.	2018	Human	52	Titanium	6, 8 months	Stevenel blue and Van Gieson picro fuchsin	Yes	BIC, MIC, CTIC
17	[69]	Cho et al.	2019	Rabbit	16	Titanium	2 weeks	modified Goldner’s Masson trichrome	No	BIC, BA
18	[70]	Choi et al.	2018	Rabbit	24	Titanium	10 days	Alizarin red; Masson-Goldner trichrome	No	BIC, BA
19	[71]	Coelho et al.	2014	Rat	40	Titanium	1, 2, 4, 8 weeks	Not stated	No	BIC, BAFO
20	[72]	Coelho et al.	2014	Dog	306	Titanium	1, 2, 4 weeks	Toluidine blue; Stevenel’s blue and van Gieson	No	BIC, BAFO
21	[73]	Cohen et al.	2016	Rabbit	40	Titanium	42 days	Toluidine blue	No	c-BIC, t-BIC
22	[74]	Dagher et al.	2014	Sheep	32	Titanium	1, 2 months	Giemsa-Paragon	No	BIC, ISB, ISA
23	[75]	de Barros et al.	2015	Dog	48	Titanium	8 weeks	Toluidine blue	No	BIC, BD, IS-BIC, HBA, IS-BC, CR, BR
24	[76]	De Barros et al.	2014	Minipig	52	Titanium	8 weeks	Stevenel’s blue and Alizarin red S	No	BIC, BD, BR-I, BR-FE, CBR
25	[77]	De Jesus et al.	2016	Dog	24	Titanium	2, 4 weeks	Toluidine blue; van Giessons pichro fuchsin	No	BIC, BD
26	[78]	De Deco et al.	2015	Rat	27	Titanium	30 days	Toluidine blue	No	BIC
27	[79]	De Val et al.	2017	Dog	80	Titanium	4, 8 weeks	Toluidine blue	No	BIC
28	[80]	Du et al.	2016	Rat	42	Titanium	3, 7, 14, 28, 56 days	methylene blue-alizarin red S	No	BIC, new BA
29	[81]	Du et al.	2016	Rat	64	Titanium	3, 7, 14, 28 days	methylene blue-alizarin red S	No	BIC, new BA
30	[82]	Dundar et al.	2018	Rat	40	Titanium	12 weeks	Toluidine blue	No	BIC
31	[83]	Elmali et al.	2020	Rabbit	24	Titanium	28 days	Toluidine blue	Yes	BIC
32	[84]	Erdogan et al.	2013	Pig	9	Titanium	8 weeks	Toluidine blue	No	BIC, BV/TV, TbTh, TbSp
33	[85]	Felgueiras et al..	2017	Rabbit	84	Titanium	4, 12, 48 weeks	Stevenel blue and van Gieson Picrofuschin	No	BIC, BA
34	[86]	Fernández-Domínguez et al.	2019	Dog	48	Titanium	3 months	picro-syrius-hematoxylin	Yes	BIC, CBL
35	[87]	Fetner et al.	2015	Dog	36	Titanium	6 months	Stevenel blue and van Gieson fuschin	No	BIC, BAFO
36	[88]	Freitas et al.	2016	Rabbit	36	Titanium	2, 6 weeks	Stevenel’s blue and Alizarin red	No	BIC, BABT, BAMA
37	[89]	Gabler et al.	2015	Rat	15	Titanium	6 weeks	Toluidine blue	No	BIC
38	[90]	Galárraga-Vinueza et al.	2020	Human	5	Titanium	5.1, 10, 15, 20.3, years	Levai–Laczko	No	BIC, BD, DL, RB
39	[91]	Galli et al.	2016	Rabbit	40	Titanium	6 weeks	Toluidine blue-pyronin	No	BIC, BA, NBA
40	[92]	Gil et al.	2016	Dog	48	Titanium	1, 6 weeks	Stevenel’s blue and van Gieson	No	BIC, BAFO
41	[93]	Gil et al.	2017	Dog	48	Titanium	3, 5 weeks	Stevenel’s blue and van Gieson	No	BIC; BAFO
42	[94]	Gil et al.	2015	Human	93	Titanium	120 days to 18 years	Toluidine blue	No	BIC, BAFO
43	[95]	Görmez et al.	2015	Pig	24	Titanium	10 weeks	Toluidine blue	Yes No	BIC, BF, RG
44	[96]	Guillaume et al.	2021	Rabbit	8	Titanium	12 weeks	van Gieson picro-fuchsin and Stevenel’s blue	No	BIC
45	[97]	Gurzawska et al.	2017	Rabbit	128	Titanium	2, 4, 6, 8 weeks	Toluidine blue	No	BIC, BD
46	[98]	Herrero-Climent et al.	2018	Minipig	48	Titanium	2, 4, 8 weeks	Toluidine blue	No	BIC, BAD
47	[99]	Hirota et al.	2014	Rabbit	12	Zirconia	12 weeks	Methylene blue and basic Fuchsin;	No	BIC, BM
48	[100]	Hong et al.	2014	Rabbit	6	Titanium	1 week	Hematoxylin and Eosin	No	BIC, BA
49	[101]	Hoornaert et al.	2020	Minipig	60	Titanium	4, 12 weeks	Hematoxylin-Eosin- Safran; Masson’s Trichrome; Toluidine blue/basic fuchsine	No	BIC
50	[102]	Janner et al.	2018	Dog	60	Titanium; Zirconia	4, 16 weeks	Toluidine blue/ McNeal combined with basic fuchsin	No	BIC, BD
51	[103]	Jimbo et al.	2014	Sheep	48	Titanium	3, 6 weeks	Stevenel’s blue	No	BIC, BAFO
52	[104]	Jimbo et al.	2014	Sheep	48	Titanium	3, 6 weeks	Toluidine blue	No	BIC, BAFO
53	[105]	Jimbo et al.	2014	Sheep	48	Not stated	3, 6 weeks	Toluidine blue	No	BIC, BAFO
54	[106]	Jimbo et al.	2014	Dog	72	Titanium	1, 5 weeks	Toluidine blue	No	BIC, BAFO
55	[107]	Kämmerer et al.	2020	Minipig	36	Titanium	2, 4, 8 weeks	Toluidine blue	No	cBIC, aBIC
56	[108]	Kim et al.	2020	Dog	12	Titanium	2, 3, 4, 6 weeks	Multiple Stain Solution (MSS)	No	BIC, BA
57	[109]	Kim et al.	2021	Rabbit	8 8	Titanium; Zirconia	4 weeks	Masson’s trichrome	No	BIC, BA
58	[110]	Kim et al.	2022	Rabbit	39	Titanium	4 weeks	Haematoxylin and Eosin	No	BIC, BA
59	[111]	Kohal et al.	2016	Human	22	Zirconia	2, 4 weeks	Toluidine blue and pararosaniline	No	BIC, BD
60	[112]	Korn et al.	2019	Rat	64	Titanium	2, 4 weeks	Masson-Goldner trichrome	No	BIC, BA/TA
61	[113]	Kubasiewicz-Ross et al.	2018	Minipig	60	Titanium; Zirconia	12 weeks	Masson–Goldner trichrome	No	BIC
62	[114]	Kuo et al.	2017	Minipig	44	Titanium; Zirconia	8, 16 weeks	Masson–Goldner trichrome	No	BIC
63	[115]	Kwak et al.	2022	Rabbit	32	Titanium	2, 4 weeks	Masson–Goldner trichrome	No	BIC
64	[116]	Lang et al.	2023	Dog	64	Titanium	2, 6 weeks	Basic fuchsin and Toluidine blue/McNeal	No	BIC
65	[117]	Lee et al.	2015	Rabbit	10	Titanium	1 week	Haematoxylin and Eosin	No	BIC, BA
66	[118]	Lee et al.	2019	Dog	54	Titanium	2, 4, 12 weeks	Masson–Goldner trichrome	No	b-MBL, l-MBL, mBIC, OIC, tBIC, mBAFO, OAFO, tBAFO
67	[119]	Lee et al.	2014	Minipig	24	Not stated	4 weeks	Haematoxylin and Eosin	Yes	BIC
68	[120]	Lee et al.	2014	Rabbit	10	Titanium	4 weeks	Haematoxylin and Eosin	Yes	BIC
69	[121]	Lopez et al.	2017	Sheep	24	Not stated	3, 6 weeks	Stevenel’s Blue and Van Gieson.	No	BIC, BAFO
70	[122]	Lyu et al.	2021	Rabbit	18	Titanium	4 weeks	Haematoxylin and Eosin	Yes No	BIC
71	[123]	Madi et al.	2016	Dog	32	Titanium	4 months	Toluidine blue	No	BIC
72	[124]	Makary et al.	2023	Human	35	Titanium	4, 6 weeks	azure II/methylene blue and fuchsine acid	No	BIC
73	[125]	Martins et al.	2018	Rabbit	30	Titanium; Zirconia	8 weeks	Toluidine blue	No	BIC, BA
74	[126]	Mehl et al.	2016	Minipig	64	Titanium	6 months	Toluidine blue	No	BIC
75	[127]	Mehl et al.	2018	Minipig	48	Titanium	6 months	Toluidine blue	No	BIC
76	[128]	Moest et al.	2014	Pig	54	Titanium	14 and 28 days	Toluidine blue	Yes No	BIC
77	[129]	Neiva et al.	2016	Dog	Not stated	Titanium	6 weeks	Stevenel’s Blue and Van Gieson	No	BAFO
78	[130]	Neldam et al.	2017	Goat	35	Titanium	20 weeks	Toluidine blue	Yes	BIC
79	[131]	Nevins et al.	2020	Human	8	Titanium	6 months	Levai–Laczko	Yes	BIC
80	[132]	Offermanns et al.	2018	Rabbit	72	Titanium	2, 12 weeks	Toluidine blue	No	BIC, BF
81	[133]	Offermanns et al.	2018	Rabbit	24	Titanium	2 weeks	Toluidine blue	No	BIC, BF
82	[134]	Pinheiro et al.	2014	Rabbit	12	Titanium	3 weeks	Stevenel’s blue and Alizarin red	No	BIC
83	[135]	Rahmati et al.	2020	Dog	36	Titanium	4 weeks	Levai–Laczko	No	BIC, BA
84	[136]	Raita et al.	2014	Rat	18	Titanium	3, 9 weeks	Methylene blue and Basic fuchsin	No	BIC, BM
85	[137]	Reinedahl et al.	2019	Rabbit	40	Titanium	8 weeks	Toluidine blue mixed with Pyronin G	No	BIC
86	[138]	Salou et al.	2015	Rabbit	16	Titanium	2, 4 weeks	methylene blue/basic fushin	No	BIC
87	[139]	Sam et al.	2020	Human	20	Titanium	8 weeks	Toluidine blue	No	BIC, BF
88	[140]	Sanz-Esporrin et al.	2021	Dog	80	Titanium	2 and 8 weeks	Levai–Laczko	No	BIC
89	[141]	Sanz-Martin et al.	2017	Dog	32	Titanium	4 and 12 weeks	Levai–Laczko	No	BIC
90	[142]	Sarendranath et al.	2015	Dog	72	Titanium	3 and 5 weeks	Stevenel’s blue and Van Geison	No	BIC, BAFO
91	[143]	Saulacic et al.	2014	Minipig	72	Zirconia	1, 2, 4, 8 weeks	Toluidine blue	No	BIC, BA
92	[144]	Schmitt et al.	2016	Dog	80	Titanium	2 and 7 days, and 6 months	Toluidine blue	No	BIC
93	[145]	Skiba et al.	2023	Human	6	Titanium	60 days	Stevenel’s blue and alizarin red	No	BIC
94	[146]	Spin-Neto et al.	2014	Human	34	Titanium	4 and 6 months	Toluidine blue	Yes	BIC, BA
95	[147]	Stocchero et al.	2018	Sheep	48	Titanium	5 and 10 weeks	Toluidine blue and pyronin G	No	tBIC, newBIC, BAFO
96	[148]	Stoc-chero et al.	2019	Sheep	40	Titanium	5 weeks	Toluidine blue and pyronin G	No	BIC, BAFO
97	[149]	Stokholm et al.	2014	Monkey	20	Titanium	3 and 6 months	Basic fuchsin and counterstaining with light green	No	BIC
98	[150]	Streckbein et al.	2014	Dog	44	Titanium	6, 12 weeks	Toluidine blue	No	BIC
99	[151]	Suaid et al.	2014	Dog	64	Titanium	12 weeks	Toluidine blue	Yes No	BD, VCBR, CR
100	[152]	Susin et al.	2019	Minipig	70	Titanium	6, 12 weeks	Sanderson’s RBS stain and acid fuchsin	No	BIC, first BIC, CBL, BDOT, BDWT
101	[153]	Thoma et al.	2015	Dog	48	Titanium; Zirconia	6 months	van Gieson	No	BIC
102	[154]	Thoma et al.	2019	Dog	30	Titanium; Zirconia	2 weeks and 6 months	van Gieson	No	BIC
103	[155]	Valles et al.	2018	Dog	60	Titanium	6 months	Levai–Laczko	No	IS–BIC, IS–BC
104	[156]	Velasco-Ortega et al.	2019	Rabbit	Not stated	Titanium	12 weeks	Levai–Laczko	No	BIC, BV/TV
105	[157]	Velasco-Ortega, E.	2021	Rabbit	20	Titanium	12 weeks	Levai–Laczko	No	BIC
106	[158]	Verket et al.	2014	Minipig	16	Titanium	6 weeks	Hematoxylin and Eosin	Yes	BIC
107	[159]	Verket et al.	2016	Minipig	16	Titanium	3 months	Hematoxylin and Eosin	Yes	BIC
108	[160]	Veronesi et al.	2017	Rabbit	14	Titanium	2, 4, 8 weeks	Toluidine blue and Fast green	No	BIC
109	[161]	Vignoletti et al.	2019	Dog	Not stated	Zirconium	2, 8 weeks	Levai–Laczko	No	BIC
110	[162]	Von Wilmowsky et al.	2016	Pig	24	Titanium	90 days	Toluidine blue	Yes No	NBH, NFB, BIC-D, BIC-L, BD-D, BD-L, BM-D, BM-L
111	[163]	Wang et al.	2016	Rabbit	24	Titanium	4, 8 weeks	Toluidine blue	No	BIC
112	[164]	Witek et al.	2020	Sheep	40	Titanium	3 and 6 weeks	Stevenel’s Blue and Van Gieson’s Picro Fuschin	No	BIC, BAFO
113	[165]	Yagi et al.	2017	Rat	36	Titanium	2, 4 weeks	Methylene blue and Basic fuchsin	No	BIC, BM
114	[166]	Yoo et al.	2015	Sheep	72	Titanium	3, 6 weeks	Stevenel’s blue and van Gieson’s picrofuchsin	No	BIC, BAFO
115	[167]	Zhou et al.	2017	Minipig	72	Titanium	3, 6, 12 weeks	Levai–Laczko	No	rB.Ar, sBV/BV, Rm.N/Im.Bd

aBIC—percentage area coverage of bone-to-implant contact within representative bone chambers, b-MBL—vertical marginal bone loss at the buccal aspect of the implant, BA—bone area, BA/TA—cancellous bone area per tissue area, BABT—bone area between threads, BAD—bone area density, BAF—bone area fraction, BAFO—bone area fraction occupancy, BAMA—bone area within mirror area, BC-BD—the bone crest and the bottom of the bone defect, BD-D—bone density defect, BD-L—bone density local bone, BDOT—bone density outside the implant threads, BDWT—bone density within the implant threads, BIC—bone to implant contact, BIC-D—bone to implant contact defect, BIC-L—bone to implant contact local bone, BF—new bone formation, BM—bone mass, BM-D—bone mineralization defect, BM-L—bone mineralization local bone, BR-FE—bone resorption around the implants- free-ends’ regions, BR-I—bone resorption around the implants-inter implant region, BV/TV—bone volume per tissue volume, c-BIC—crestal bone-to-implant contact, cBIC—percentage area coverage of cortical bone-to-implant, CBL—crestal bone loss, Ct.Th—cortical bone thickness, CTIC—connective tissue-to-implant contact, CBL—vestibular and lingual crestal bone loss, CBR—crestal bone resorption between implants, CR—crestal resorption, DL—defect length, l-MBL—the vertical marginal bone loss at the lingual aspect of the implant, imp-BC—the implant surface and the alveolar bone crest, HBA—horizontal bone apposition, ISA—total implant surface available, ISB—Linear surface of the implant in direct contact with bone, IS-BC—implant shoulder to bone crest level, IS-BD—the implant shoulder and bottom of the bone defect, IS-BIC—implant shoulder to first bone-to-implant contact, LB—area of lamellar bone interthread regions/total interthread area, mBAFO—mineralized bone area fraction occupied, mBIC—mineralized bone-to-implant contact, MIC—marrow to implant contact, NBA—new bone area, NBF—newly formed bone, NBH—new bone height, OAFO—osteoid area fraction occupied, OIC—osteoid-to-implant contact, PIBA—peri-implant bone-area, rB.Ar—area of remodeled bone, RB—residual bone, RG—area residual graft particles interthread regions/total interthread area, Rm.N/Im.Bd—number of remodeling sites at the implant interface, sBV/BV—percentage of remodeled bone, STM—area of soft tissue interthread regions/total interthread area, tBAFO—total bone area fraction occupied, tBIC—total bone to implant contact, TbTh—trabecular thickness, TbSp—trabecular separation, VCBR—vertical crestal bone resorption, WB—area of woven bone interthread regions/total interthread area.

**Table 2:** Regions of interest (ROIs) and the evaluated histomorphometric parameters.

№	Reference	Authors	Year	Region of Interest (ROI)	Histomorphometric Parameter
1	[57]	Al-Omari et al.	2020	The regions around the dental implant—between 0 μm and 1000 μm away from the implant surface horizontally and from the implant shoulder up to 2500 μm below the implant platform longitudinally. The ROIs for Ct.Th were at 250 μm, 500 μm, and 750 μm away from the implant surface at the study endpoint.	**BIC** **BAF** **Ct.Th**
2	[58]	Alshehri et al.	2019	A rectangle with a length of 5 mm, equal to the porous portion of the implant, and a height of 0.5 mm (adjacent to the implant surface).	**PIBA**
3	[60]	Beutel et al.	2016	ROI 1—the region of cortical bone. ROI 2—the region of trabecular bone.	**BIC** **BAFO**
4	[68]	Chiu et al.	2018	Total implant length; the first four threads at the most coronal aspect, starting from the grafted area (4T); and the two threads (2T) apical to 4T.	**BIC** **MIC** **CTIC**
5	[70]	Cho et al.	2019	The region from the bone crest to 2 mm in depth.	**BIC** **BA**
6	[72]	Coelho et al.	2014	For BIC—the total implant perimeter. For BAFO—the total area between threads.	**BIC** **BAFO**
7	[75]	de Barros et al.	2015	ROIs—for each section on each implant side (left and right). In each case, measurements were performed in the ROI directly adjacent to the implant (adjacent ROI) and one of identical shape, neighboring the adjacent ROI, at a further distance from the implant (distant ROI). Each ROI had a rectangular shape, and the adjacent ROIs included various peri-implant defects (0.5, 1.0, or 2.0 mm in width and 5 mm in distance from the implant shoulder).	**BD** **BIC**
8	[76]	De Barros et al.	2014	The region between adjacent implants, using a predetermined rectangle as a frame to select the areas to be assessed.	**SAQW**
9	[80]	Du et al.	2015	For BIC—the total implant perimeter. For BA—the total area between threads.	**BIC** **BA**
10	[81]	Du et al.	2016	For BIC—the total implant perimeter. For BA—the total area between threads.	**BIC** **BA**
11	[82]	Dundar et al.	2018	The total implant perimeter.	**BIC**
12	[83]	Elmali et al.	2020	From the implant shoulder to the end of the gap (4 mm).	**BIC** **NBF**
13	[85]	Felgueiras et al.	2017	The area delineated by a circle drawn at a distance of 100 µm from the implant.	**BIC** **BA**
14	[86]	Fernández-Domínguez et al.	2019	For CBL—from the implant platform to the first point of bone-to-implant contact. For BIC—two regions (lingual and buccal), from the implant shoulder to the apical part	**CBL** **BIC**
15	[87]	Fetner et al.	2015	For BIC—the total implant perimeter. For BA—the total area between threads.	**BIC** **BAFO**
16	[88]	Freitas et al.	2016	ROI—from the first thread down toward the fourth one, both medially and distally. For BIC and BABT—the area between threads. For BAMA—the area outside the threads. This mirror area is a symmetrical area to the trapezoid between two threads, sharing its larger base.	**BIC** **BABT** **BAMA**
17	[89]	Gabler et al.	2015	The length of the shell area of each implant—only those parts of the implant that were completely surrounded by bone tissue.	**BIC**
18	[90]	Galárraga-Vinueza et al.	2020	The residual bone was divided into 500 μm-wide horizontal zones following the long axis of the implant.	**DL** **RB ** **BIC** **B.Ar/T.Ar**
19	[92]	Gil et al.	2016	For BIC—the total implant perimeter. For BAFO—the total area between threads.	**BIC** **BAFO**
20	[97]	Gurzawska et al.	2017	For BIC—a zone from the upper level of the cortical bone to half of the length (between the second and third thread). For BD—the region within two threads in the cortical bone.	**BIC** **BD**
21	[99]	Hirota et al.	2014	The total implant perimeter.	**BIC** **BM**
22	[111]	Kohal et al.	2016	For BIC—from the first bone-to-implant contact point to the apical part of the implant on both sides. For BA—the area within the implant threads on both sides of each implant.	**BIC** **BA**
23	[117]	Lee et al.	2015	The range of 2 mm below the upper bone crest.	**BIC** **BA**
24	[118]	Lee et al.	2019	The peri-implant area, located between 3 and 6 mm below the implant shoulder.	**b-MBL** **l-MBL** **mBIC** **OIC** **tBIC** **mBAFO** **OAFO** **tBAFO**
25	[128]	Moest et al.	2014	Four ROIs—two in the crestal and two in the apical implant region.	**BIC**
26	[130]	Neldam et al.	2017	The peri-implant bone. The implant was divided into two vertical zones: the micro thread area and the macro thread area	**BIC**
27	[132]	Offermanns et al.	2018	ROIs at 0–250 μm and 250–500 μm distances from the implant surfaces.	**BIC** **BF**
28	[133]	Offermanns et al.	2018	ROIs at 0–250 μm (inside the thread of the implant) and 250–500 μm (outside the thread of the implant) distances.	**BIC** **BF**
29	[135]	Rahmati et al.	2020	Around the bone chamber implant (a rectangular ROI).	**BIC** **BA**
30	[136]	Raita et al.	2014	The total of both the mesial and distal triangular areas formed by the threads.	**BIC** **BM**
31	[139]	Sam et al.	2020	For BIC—the total length implant surface. For NBF—the total area of the implant chamber.	**BIC** **NBF**
32	[140]	Sanz-Esporrin et al.	2021	The most coronal portion (3 mm) and the entire surface (6 coronal mm) of the implant.	**BIC**
33	[142]	Saulacic et al.	2014	The surface of the implant groove and the extension of the outer implant diameter.	**BIC** **BA**
34	[144]	Schmitt et al.	2016	Regions of interest: ROIs 1 and 4 in the gap of the middle part of the implant, ROIs 2 and 3 in the threaded apical part, and ROI 5 at the tip of the implant.	**BIC**
35	[145]	Skiba et al.	2023	The whole implant perimeter.	**BIC**
36	[146]	Spin-Neto et al.	2014	On each side of the mini-implant, three equally sized regions (length: 650 lm; area: 3500 lm2) were traced, representing the “coronal” (R1), “middle” (R2), and “apical” (R3) portions of the mini-implant (corresponding to threads 1–2, 7–8, and 13–14 from the head of the screw, respectively).	**BA** ** BIC**
37	[150]	Streckbein et al.	2014	The total implant surface area.	**BICRs**
38	[151]	Suaid et al.	2014	Within a rectangle that comprises the region of the defect, 1.0 mm wide and 3 mm in length (total area obtained), from the implant shoulder (adjacent areas) and also within another in a mirror image of the first areas (distant areas).	**BD**
39	[153]	Thoma et al.	2015	The ROI was defined with a length of 4 mm in the center on the buccal and lingual sides of each implant.	**BIC**
40	[154]	Thoma et al.	2019	The ROI was defined with a length of 4 mm in the center on the buccal and lingual sides of each implant.	**BIC**
41	[161]	Vignoletti et al.	2019	At the most coronal portion (3 mm) as well as the overall dimension of the implant.	**BIC**
42	[167]	Zhou et al.	2017	A 200 µm-wide zone parallel to the implant surface from the implant shoulder downward.	**rB.Ar** **sBV/BV** **B.Ar** **Rm.N/Im.Bd**

BA—bone area, BABT—bone area between threads, BAF—bone area fraction, BAFO—bone area fraction occupancy, BAMA—bone area within mirror area, B.Ar—total bone area, B.Ar/T.Ar—bone density, BD—bone density, BF—new bone formation, BIC—bone to implant contact, BICRs—bone to implant contacts, BM—bone mass, b-MBL—vertical marginal bone loss at the buccal aspect of the implant, CBL—crestal bone loss, CTIC—connective tissue to implant contact, CT.Th—cortical bone thickness, DL—defect length, l-MBL—the vertical marginal bone loss at the lingual aspect of the implant, mBAFO—mineralized bone area fraction occupied, mBIC—mineralized bone to implant contact, MIC—marrow to implant contact, NBF—newly formed bone, OAFO—osteoid area fraction occupied, OIC—osteoid to implant contact, PIBA—peri-implant bone area, RB—residual bone, rB.Ar—area of remodeled bone, Rm.N/Im.Bd—number of remodeling sites at the implant interface, sBV/BV—percentage of remodeled bone, tBAFO—total bone area fraction occupied, tBIC—total bone to implant contact.

## Data Availability

The original data presented in the study are openly available in the Open Science Framework (OSF) and can be found at https://archive.org/details/osf-registrations-9b8yx-v1 (accessed on 12 July 2024).
